# 3-(4-Pyrid­yl)benzoic acid

**DOI:** 10.1107/S1600536809015530

**Published:** 2009-05-14

**Authors:** Jianxin Xing

**Affiliations:** a Department of Biology, Dezhou University, Dezhou Shandong 253023, People’s Republic of China

## Abstract

The mol­ecule of the title compound, C_12_H_9_NO_2_, is not planar, the benzene and pyridine rings making a dihedral angle of 32.14 (7)°. The carb­oxy group is slightly twisted with respect to the benzene ring by 11.95 (10)°. In the crystal structure, inter­molecular O—H⋯N hydrogen bonds link neighboring mol­ecules into infinite chains along the *c* axis.

## Related literature

For coordination polymers with pyridine carboxyl­ate, see: Lu & Luck (2003 ▶); Luo *et al.* (2007 ▶).
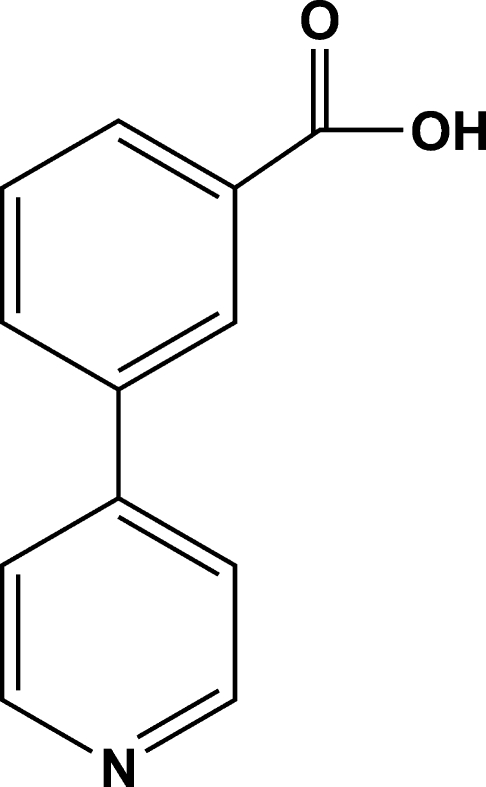


## Experimental

### 

#### Crystal data

C_12_H_9_NO_2_
*M* *_r_* = 199.20
Orthorhombic, 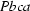

*a* = 13.839 (3) Å
*b* = 7.013 (7) Å
*c* = 19.469 (10) Å
*V* = 1890 (2) Å^3^
*Z* = 8
Mo *K*α radiation
μ = 0.10 mm^−1^
*T* = 296 K
0.33 × 0.25 × 0.20 mm

#### Data collection

Bruker APEXII CCD area-detector diffractometer
Absorption correction: multi-scan (*SADABS*; Bruker, 2005 ▶) *T*_min_ = 0.958, *T*_max_ = 0.979
11481 measured reflections
2365 independent reflections
1480 reflections with *I* > 2σ(*I*)
*R*_int_ = 0.041

#### Refinement

*R*[*F* ^2^ > 2σ(*F* ^2^)] = 0.045
*wR*(*F* ^2^) = 0.135
*S* = 1.03
2365 reflections
137 parameters
H-atom parameters constrained
Δρ_max_ = 0.24 e Å^−3^
Δρ_min_ = −0.19 e Å^−3^

### 

Data collection: *APEX2* (Bruker, 2005 ▶); cell refinement: *SAINT* (Bruker, 2005 ▶); data reduction: *SAINT*; program(s) used to solve structure: *SIR97* (Altomare *et al.*, 1999 ▶); program(s) used to refine structure: *SHELXL97* (Sheldrick, 2008 ▶); molecular graphics: *DIAMOND* (Brandenburg & Putz, 1999 ▶); software used to prepare material for publication: *WinGX* (Farrugia, 1999 ▶).

## Supplementary Material

Crystal structure: contains datablocks global, I. DOI: 10.1107/S1600536809015530/dn2447sup1.cif

Structure factors: contains datablocks I. DOI: 10.1107/S1600536809015530/dn2447Isup2.hkl

Additional supplementary materials:  crystallographic information; 3D view; checkCIF report

## Figures and Tables

**Table 1:** Hydrogen-bond geometry (Å, °)

*D*—H⋯*A*	*D*—H	H⋯*A*	*D*⋯*A*	*D*—H⋯*A*
O2—H2⋯N1^i^	0.82	1.83	2.6526 (18)	178

Symmetry code: (i) 
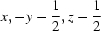
.

**Fig. 1.**
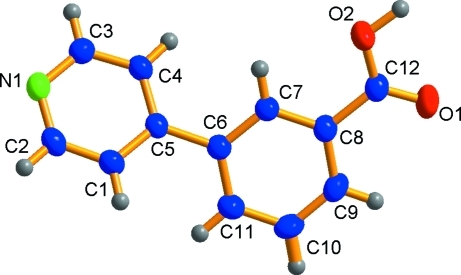
Molecular structure of the title compound with the atom labeling scheme. Displacement ellipsoids are drawn at the 50% probalility level. H atoms are represented as small spheres of arbitrary radii.

**Fig. 2.**
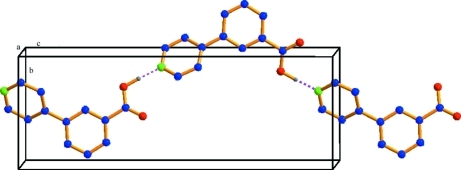
Partial packing view showing the formation of infinite chain through the O-H···N hydrogen bondings. H bonds are shown as dashed lines. H atoms not involved in hydrogen bondings have been omitted for clarity.

## supplementary crystallographic information

### Comment

As part of an ongoing investigation into coordination polymer with pyridine carboxylate (Lu *et al.*, 2003; Luo *et al.*, 2007), the crystal structure of the title compound is presented here.

The molecule of the title compound, C_12_H_9_NO_2_, is not planar, the phenyl and the pyridine rings make a dihedral angle of 32.14 (7)° (Fig. 1). The acetic group is slightly twisted with respect to the phenyl ring by 11.95 (10)°. In the crystal structure, intermolecular O—H···N hydrogen bonds link neighboring molecules into infinite chains along the c axis (Table 1, Fig. 2).

### Experimental

Commercially available 3-Pyrid-4-ylbenzoic acid was further purified by repeated recrystallization anhydrous ethanol from. Single crystals suitable for X-ray analysis were grown by slow evaporation of an anhydrous ethanol solution at room temperature.

### Refinement

All H atoms attached to C atoms and O atom were fixed geometrically and treated as riding with C—H = 0.93 Å and O—H = 0.82 Å with U_iso_(H) = 1.2U_eq_(C) or U_iso_(H) = 1.5U_eq_(O).

### Crystal data

**Table:**

C_12_H_9_NO_2_	*F*(000) = 832
*M**_r_* = 199.20	*D*_x_ = 1.400 Mg m^−^^3^
Orthorhombic, *P**b**c**a*	Mo *K*α radiation, λ = 0.71069 Å
Hall symbol: -P 2ac 2ab	Cell parameters from 1695 reflections
*a* = 13.839 (3) Å	θ = 2.6–24.3°
*b* = 7.013 (7) Å	µ = 0.10 mm^−^^1^
*c* = 19.469 (10) Å	*T* = 296 K
*V* = 1890 (2) Å^3^	Block, colorless
*Z* = 8	0.33 × 0.25 × 0.20 mm

### Data collection

**Table:**

Bruker APEX2 CCD area-detector diffractometer	2365 independent reflections
Radiation source: fine-focus sealed tube	1480 reflections with *I* > 2σ(*I*)
graphite	*R*_int_ = 0.041
φ and ω scans	θ_max_ = 28.5°, θ_min_ = 2.1°
Absorption correction: multi-scan (*SADABS*; Bruker, 2005)	*h* = −12→18
*T*_min_ = 0.958, *T*_max_ = 0.979	*k* = −9→8
11481 measured reflections	*l* = −25→26

### Refinement

**Table:**

Refinement on *F*^2^	Primary atom site location: structure-invariant direct methods
Least-squares matrix: full	Secondary atom site location: difference Fourier map
*R*[*F*^2^ > 2σ(*F*^2^)] = 0.045	Hydrogen site location: inferred from neighbouring sites
*wR*(*F*^2^) = 0.135	H-atom parameters constrained
*S* = 1.02	*w* = 1/[σ^2^(*F*_o_^2^) + (0.0645*P*)^2^ + 0.2238*P*] where *P* = (*F*_o_^2^ + 2*F*_c_^2^)/3
2365 reflections	(Δ/σ)_max_ = 0.001
137 parameters	Δρ_max_ = 0.24 e Å^−^^3^
0 restraints	Δρ_min_ = −0.18 e Å^−^^3^

### Special details

**Table:**

Geometry. All esds (except the esd in the dihedral angle between two l.s. planes) are estimated using the full covariance matrix. The cell esds are taken into account individually in the estimation of esds in distances, angles and torsion angles; correlations between esds in cell parameters are only used when they are defined by crystal symmetry. An approximate (isotropic) treatment of cell esds is used for estimating esds involving l.s. planes.
Refinement. Refinement of *F*^2^ against ALL reflections. The weighted *R*-factor *wR* and goodness of fit *S* are based on *F*^2^, conventional *R*-factors *R* are based on *F*, with *F* set to zero for negative *F*^2^. The threshold expression of *F*^2^ > σ(*F*^2^) is used only for calculating *R*-factors(gt) *etc*. and is not relevant to the choice of reflections for refinement. *R*-factors based on *F*^2^ are statistically about twice as large as those based on *F*, and *R*- factors based on ALL data will be even larger.

### Fractional atomic coordinates and isotropic or equivalent isotropic displacement parameters (Å^2^)

**Table:**

	*x*	*y*	*z*	*U*_iso_*/*U*_eq_	
O1	0.40429 (11)	0.07701 (18)	0.11778 (6)	0.0584 (4)	
O2	0.36926 (10)	−0.18026 (17)	0.17987 (6)	0.0522 (4)	
H2	0.3707	−0.2322	0.1422	0.078*	
N1	0.37092 (10)	−0.1441 (2)	0.55920 (6)	0.0404 (4)	
C1	0.32413 (12)	0.1110 (2)	0.48533 (8)	0.0394 (4)	
H1	0.2910	0.2254	0.4801	0.047*	
C2	0.32519 (12)	0.0204 (2)	0.54811 (8)	0.0409 (4)	
H2A	0.2924	0.0767	0.5845	0.049*	
C3	0.41717 (12)	−0.2214 (2)	0.50565 (8)	0.0412 (4)	
H3	0.4495	−0.3362	0.5123	0.049*	
C4	0.41965 (13)	−0.1406 (2)	0.44148 (8)	0.0384 (4)	
H4	0.4527	−0.2007	0.4059	0.046*	
C5	0.37253 (11)	0.0313 (2)	0.42988 (7)	0.0333 (4)	
C6	0.37464 (11)	0.1281 (2)	0.36208 (7)	0.0351 (4)	
C7	0.37848 (12)	0.0237 (2)	0.30137 (8)	0.0367 (4)	
H7	0.3784	−0.1088	0.3034	0.044*	
C8	0.38238 (11)	0.1135 (2)	0.23794 (8)	0.0370 (4)	
C9	0.38217 (13)	0.3104 (2)	0.23504 (9)	0.0449 (4)	
H9	0.3859	0.3718	0.1928	0.054*	
C10	0.37647 (14)	0.4159 (2)	0.29454 (9)	0.0517 (5)	
H10	0.3752	0.5484	0.2922	0.062*	
C11	0.37260 (13)	0.3262 (2)	0.35748 (9)	0.0449 (4)	
H11	0.3686	0.3989	0.3973	0.054*	
C12	0.38692 (12)	0.0033 (2)	0.17260 (8)	0.0405 (4)	

### Atomic displacement parameters (Å^2^)

**Table:**

	*U*^11^	*U*^22^	*U*^33^	*U*^12^	*U*^13^	*U*^23^
O1	0.0891 (11)	0.0554 (8)	0.0308 (7)	−0.0103 (7)	0.0039 (6)	0.0069 (6)
O2	0.0824 (10)	0.0435 (7)	0.0306 (6)	−0.0105 (7)	0.0057 (6)	−0.0034 (5)
N1	0.0462 (9)	0.0433 (8)	0.0316 (7)	−0.0036 (6)	−0.0016 (6)	0.0014 (6)
C1	0.0434 (10)	0.0387 (9)	0.0361 (9)	0.0052 (7)	0.0005 (7)	−0.0049 (7)
C2	0.0436 (10)	0.0468 (10)	0.0322 (9)	−0.0013 (8)	0.0036 (7)	−0.0073 (7)
C3	0.0483 (10)	0.0375 (8)	0.0377 (9)	0.0036 (8)	−0.0022 (7)	0.0007 (7)
C4	0.0460 (10)	0.0373 (9)	0.0319 (8)	0.0016 (7)	0.0039 (7)	−0.0037 (7)
C5	0.0374 (8)	0.0340 (8)	0.0285 (8)	−0.0034 (7)	−0.0011 (6)	−0.0023 (6)
C6	0.0381 (9)	0.0342 (8)	0.0330 (8)	−0.0005 (7)	0.0009 (7)	0.0000 (6)
C7	0.0452 (9)	0.0319 (8)	0.0330 (8)	−0.0011 (7)	0.0011 (7)	0.0008 (6)
C8	0.0406 (9)	0.0385 (9)	0.0320 (8)	−0.0026 (7)	−0.0008 (7)	0.0013 (6)
C9	0.0567 (11)	0.0409 (10)	0.0370 (9)	−0.0037 (8)	−0.0058 (8)	0.0095 (7)
C10	0.0732 (14)	0.0300 (9)	0.0519 (11)	0.0004 (9)	−0.0051 (9)	0.0038 (8)
C11	0.0590 (11)	0.0359 (9)	0.0399 (9)	0.0018 (8)	−0.0004 (8)	−0.0047 (7)
C12	0.0468 (10)	0.0423 (10)	0.0324 (9)	−0.0017 (7)	−0.0010 (7)	0.0035 (7)

### Geometric parameters (Å, °)

**Table:**

O1—C12	1.2102 (18)	C5—C6	1.485 (2)
O2—C12	1.318 (2)	C6—C7	1.391 (2)
O2—H2	0.8200	C6—C11	1.393 (2)
N1—C2	1.333 (2)	C7—C8	1.387 (2)
N1—C3	1.338 (2)	C7—H7	0.9300
C1—C2	1.378 (2)	C8—C9	1.382 (2)
C1—C5	1.388 (2)	C8—C12	1.490 (2)
C1—H1	0.9300	C9—C10	1.377 (2)
C2—H2A	0.9300	C9—H9	0.9300
C3—C4	1.372 (2)	C10—C11	1.379 (2)
C3—H3	0.9300	C10—H10	0.9300
C4—C5	1.389 (2)	C11—H11	0.9300
C4—H4	0.9300		
			
C12—O2—H2	109.5	C11—C6—C5	120.85 (13)
C2—N1—C3	116.83 (14)	C8—C7—C6	121.25 (15)
C2—C1—C5	119.96 (15)	C8—C7—H7	119.4
C2—C1—H1	120.0	C6—C7—H7	119.4
C5—C1—H1	120.0	C9—C8—C7	119.33 (15)
N1—C2—C1	123.21 (15)	C9—C8—C12	118.92 (14)
N1—C2—H2A	118.4	C7—C8—C12	121.75 (15)
C1—C2—H2A	118.4	C10—C9—C8	120.19 (15)
N1—C3—C4	123.66 (16)	C10—C9—H9	119.9
N1—C3—H3	118.2	C8—C9—H9	119.9
C4—C3—H3	118.2	C9—C10—C11	120.32 (16)
C3—C4—C5	119.64 (15)	C9—C10—H10	119.8
C3—C4—H4	120.2	C11—C10—H10	119.8
C5—C4—H4	120.2	C10—C11—C6	120.77 (15)
C1—C5—C4	116.70 (14)	C10—C11—H11	119.6
C1—C5—C6	121.14 (14)	C6—C11—H11	119.6
C4—C5—C6	122.15 (14)	O1—C12—O2	123.30 (16)
C7—C6—C11	118.12 (14)	O1—C12—C8	122.67 (16)
C7—C6—C5	121.03 (14)	O2—C12—C8	114.03 (14)

### Hydrogen-bond geometry (Å, °)

**Table:**

*D*—H···*A*	*D*—H	H···*A*	*D*···*A*	*D*—H···*A*
O2—H2···N1^i^	0.82	1.83	2.6526 (18)	178

Symmetry codes: (i) *x*, −*y*−1/2, *z*−1/2.
